# Innovative Image Processing Method to Improve Autofocusing Accuracy

**DOI:** 10.3390/s22135058

**Published:** 2022-07-05

**Authors:** Chien-Sheng Liu, Ho-Da Tu

**Affiliations:** 1 Department of Mechanical Engineering, National Cheng Kung University, Tainan 70101, Taiwan; n16084721@gs.ncku.edu.tw; 2 Academy of Innovative Semiconductor and Sustainable Manufacturing, National Cheng Kung University, Tainan 70101, Taiwan

**Keywords:** geometric fluctuations, algorithm, autofocusing, accuracy, image processing

## Abstract

For automated optical inspection, autofocusing microscopes play an important role in capturing clear images of the measured object. At present, the image processing part of optics-based autofocusing microscopes often has various factors, which makes it impossible to describe the image information of the semicircular (or elliptical) spot with a simple circle-finding method. Accordingly, this study has developed a novel algorithm that can quickly calculate the ideal center of the elliptical spot and effectively compensate the linearity of the focusing characteristic curve. A prototype model was used to characterize and verify the proposed algorithm. The experimental results show that by using the proposed algorithm, the autofocusing accuracy can be effectively improved to less than 1.5 μm.

## 1. Introduction

Automated optical inspection (AOI) has played a large role in manufacturing factories since it is able to provide accurate measurements and fast results for the inspection of defects [1,2,3]. Taking microscopic images of products and focusing is a fundamental function required for measuring and analyzing small defects [4]. During the capture of the microscopic images, focus drifts are often caused by environmental noises and result in blurred microscopic image [5]. Currently, manual focusing is a solution, but it is impractical, especially for large products due to its inaccuracy and being time-consuming. Hence, the autofocusing function is a solution with potential. In several steps prior to AOI processing, the image must be in focus, including image processing, image locating, and pattern matching [6,7]. Another application of the autofocusing function is high precision laser printing and laser machining [8]. Through the function, the laser energy can be focused on the machining areas. Therefore, many kinds of autofocusing devices and methods have been designed and proposed in the literature to realize automated capture for microscopic images and microscale fabrication using AOI, precise laser printing, and laser machining [9,10,11,12,13,14,15,16].

As introduced in [16,17], existing autofocusing technologies can be broadly classified as two kinds: passive control and active control. The autofocusing technologies with passive control are based on optimization of the image sharpness at different axial positions and find the real focal point via developed search algorithm. They are also called image-based autofocusing technologies, and they are robust and reliable. However, their main disadvantages are that they are time-consuming due to their heavy data processing [17,18,19] and that they might cause a false local maxima/minima [20].

The autofocusing technologies with active control are based on the assistance of an extra optical sensor, so they are called optics-based autofocusing technologies. Their main advantages are being highly accurate and time-saving in comparison with passive control autofocusing technologies [17,21]. As a result, autofocusing technologies with active control are popularly used in many AOI applications, precise laser printing, and laser machining with real-time and precise measurements [22,23,24,25,26,27,28,29,30,31,32]. As for the demand of critical requirements, the autofocusing technologies with active control are required to further improve their autofocusing accuracy. Accordingly, the purpose of the present study is to effectively improve the autofocusing accuracy of the autofocusing technologies with active control by using a novel algorithm.

To the best of our knowledge, WDI Wise Device Inc. has dominated the market of commercial autofocusing technologies with active control in recent ten years. References [16,33] described the detailed design of this autofocusing technology and a laser diode used as a light source. Using the centroids of the laser spots as feedback, the autofocusing function can be performed. However, the geometric laser beam variations or fluctuations with time degrade its autofocusing accuracy [34]. Therefore, in order to overcome this issue and improve the autofocusing accuracy, in general, two methods can be used. One method is to design and manufacture stable laser light sources [35,36,37], and the other is to suppress the effects of laser fluctuations computationally [2,38,39]. The latter is the focus and aim of this study.

**Figure 1:**
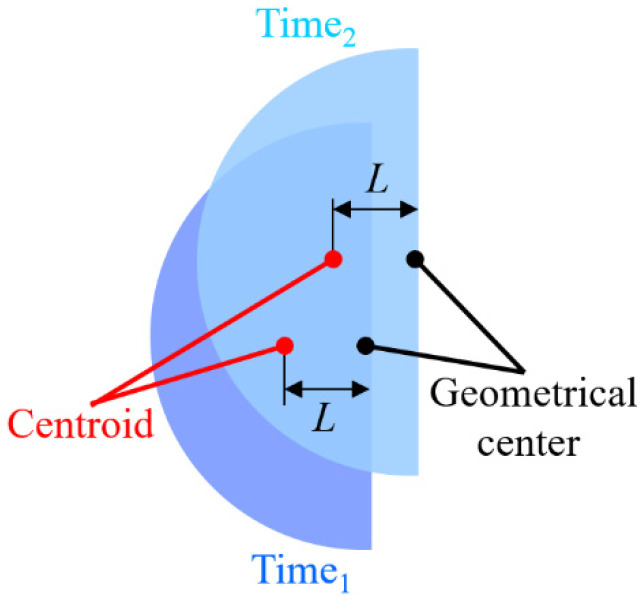
Definition of *L* between centroid and geometric center of captured laser spot images.

Hereafter, some literature has been reviewed for diminishing the effects of laser fluctuations. In our published paper, a modified autofocusing algorithm was developed, and the distance *L* between the centroid and geometric center of laser spot image is used as the modified feedback signal [34], as shown in Figure 1, but this algorithm does not consider real geometric distortion of the laser beam. In our other previous studies [2,38,39], the autofocusing accuracy has further improved by using a motor and a rotating diffuser element, but this method increases the hardware cost. So, the accuracy of the focusing characteristic curve of the microscope is further enhanced by utilizing a proposed novel algorithm to effectively suppress the effects of geometric distortions in the current study. The feasibility of the proposed novel algorithm and prototype model have been demonstrated by using a series of experiments.

## 2. Adopted Autofocusing Structure and Experimental Setup

### 2.1. Adopted Autofocusing Structure

**Figure 2:**
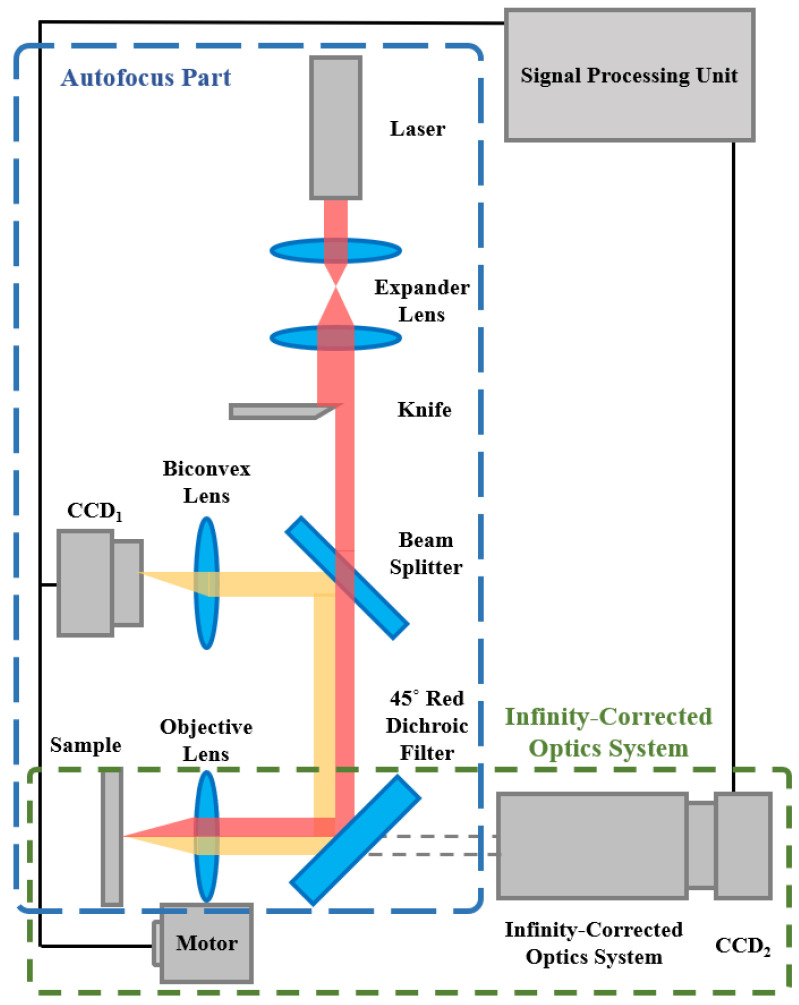
Structure of adopted autofocusing microscope.

Figure 2 illustrates the basic autofocusing structure adopted in this study. In our previous studies, we identified the detailed design parameters of the autofocusing microscope [2,34]. To verify the performance of the proposed novel algorithm, a duplicate of the autofocusing microscope was used. As shown, the adopted autofocusing structure can be divided into the autofocus part and the infinity-corrected optics system part. The autofocusing function is operated in the autofocus part. A real-time microscopic image of the sample is obtained using the infinity-corrected and coaxial optics system.

**Figure 3:**
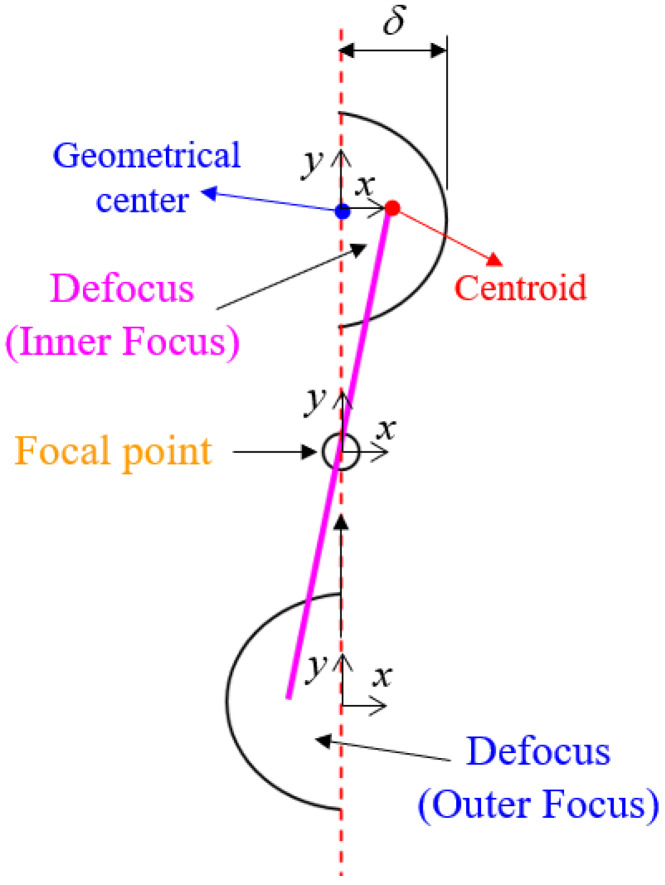
Relation between *L* and defocus distance *δ*.

According to our previous study [34], the distance L between the centroid and geometric center of the captured laser spot image on CCD_1_ changes linearly with the defocus distance *δ* (or axis move), as shown in Figure 3. A self-development algorithm is used to calculate and measure the variation of distance *L* (see Section 3). To achieve autofocusing function, the measured distance *L* was used as a position feedback signal to move the objective lens in real time by a precise motor (PI UPL120, unidirectional repeatability to 0.05 µm). Table 1 lists the selected values of each design parameter for the proposed prototype.

### 2.2. Experimental Setup of Prototype Autofocusing Microscope

**Figure 4:**
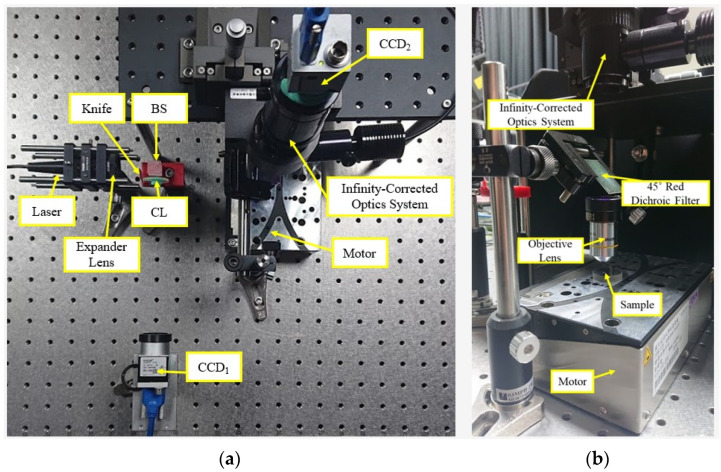
(**a**) Photograph of laboratory-built prototype; (**b**) infinity-corrected optics system part.

**Figure 5:**
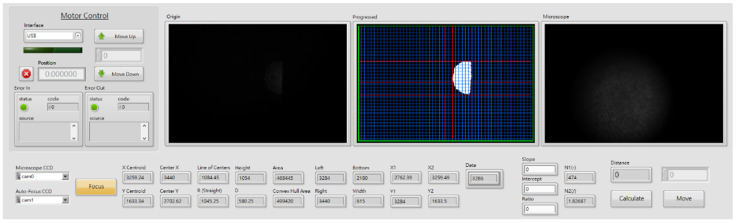
Proposed HMI.

The performance of the proposed algorithm was demonstrated by fabricating a prototype model of autofocusing microscope. The proposed algorithm was integrated into a self-development human machine interface (HMI) in commercial software of LabVIEW. The LabVIEW implementation includes the following functions: for the image processing, converting to a more ideal value of output format to enhance contrast and brightness in analysis data of image processing, and using the gray morphology function to adjust the speckle shape captured by CCD_1_. The function “clamp” measures the side edge of the speckle including top and bottom. Finally, the program in LabVIEW also captures the centroid and gravity center of the laser spot image and executes the defocus distance calculation analysis. Figure 4 shows the photograph of the prototype model of the autofocusing microscope. Figure 5 shows the proposed HMI. As shown in Figure 5, the left, middle, and right images present the original laser spot image captured by the CCD_1_, processed laser spot image, and real-time image of the sample, respectively.

## 3. Proposed Algorithm

### 3.1. Proposed Ellipse Spot Compensation Algorithm

In an ideal case, the adopted autofocusing microscope conventionally utilized a knife (see Figure 2) to produce a reflective semicircular laser spot to measure the defocus direction and distance *δ*. However, in the real case, the captured laser spot image is likely an ellipse and not an ideal semicircular geometry. The ellipse shape may be caused by assembly errors of the instrument, variable reflection characteristics, or a tilt of the sample, and so on [40]. Consequently, an ellipse spot compensation algorithm is presented in this study, which uses the boundary information of the original laser spot image captured by CCD_1_ to efficiently overcome this issue.

**Figure 6:**
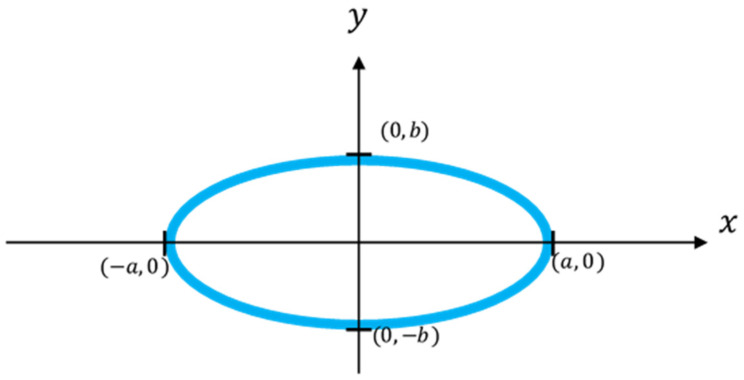
An ellipse centered at the origin.

First, according to the definition of Figure 6 and the ellipse’s equation, the equation of the left and right semicircles is obtained as:

(1) $$x=\pm \frac{a}{b}\sqrt{b^{2}-y^{2}}$$

**Figure 7:**
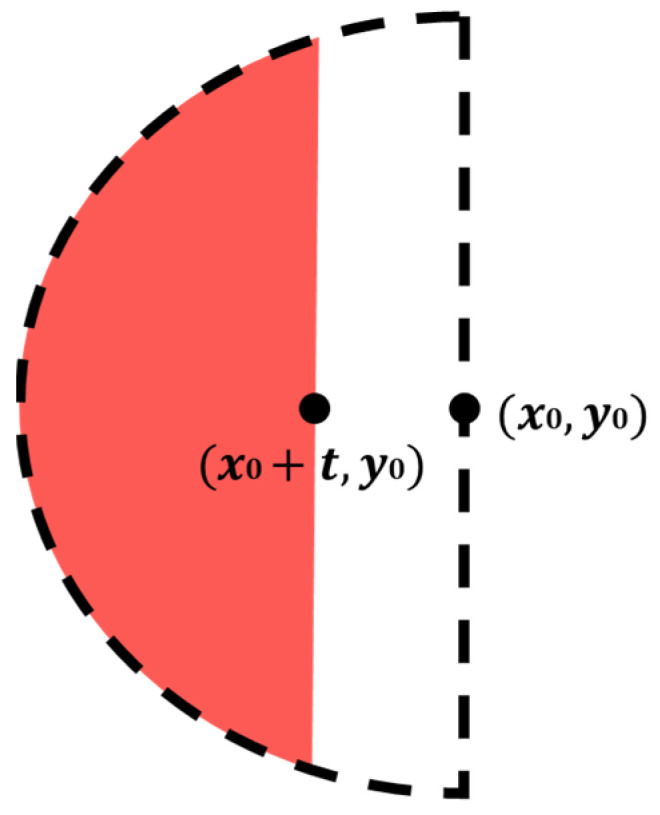
Compensation of ellipse spot image.

Assume that an original laser spot image captured by CCD_1_ is shown in the red part of Figure 7; thus the midpoint of its chord is (*x*_0_ + *t*, *y*_0_), the ideal semi-ellipse is the black dashed part in Figure 7, and its geometrical center is (*x*_0_, *y*_0_). Taking the geometrical center of the ellipse into Equation (1), Equation (2) can be obtained:

(2) $$x_{0}=\pm \frac{a}{b}\sqrt{b^{2}-y_{0}{}^{2}}$$

Take two points (*x*_1_, *y*_1_) and (*x*_2_, *y*_2_) of any asymmetric points on the arc of the captured laser spot image, and put them into Equation (2) and solve the equations to obtain *a* and *b*:

(3) $$a=\frac{\sqrt{{\left(x_{2}y_{1}\right)}^{2}-{\left(x_{1}y_{2}\right)}^{2}}}{\sqrt{y_{1}^{2}-y_{2}^{2}}}$$

(4) $$b=\sqrt{\frac{\left(x_{1}y_{2}+x_{2}y_{1}\right)\left(x_{1}y_{2}-x_{2}y_{1}\right)}{\left(x_{1}+x_{2}\right)\left(x_{1}-x_{2}\right)}}$$

**Figure 8:**
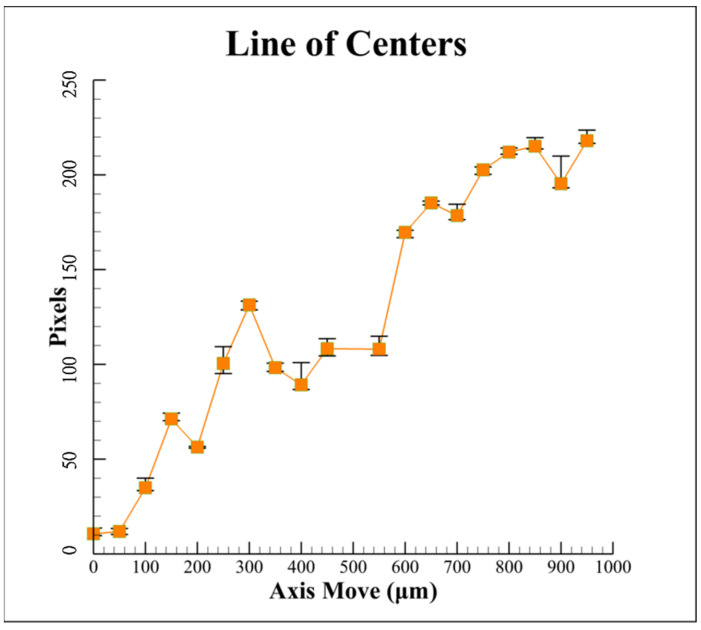
Measurement results of proposed algorithm for variation of distance *L* between centroid and geometric center with defocus distance.

From Equations (1)–(4), the ellipse equations can be used to solve for the geometric center position. Finally, the centroid of the laser spot image can be obtained through the built-in function of LabView. Finally, the distance *L* between the centroid and geometric center of the captured laser spot image on CCD_1_ was obtained. Figure 8 illustrates the calculated and measured results for the distance *L* of the image with the defocus distance by using the proposed ellipse spot compensation algorithm. Using the measurement results, it has been determined that the linearity (R2) of the proposed distance *L* vs. defocus distance curve is approximately 91.77%, which is not in good agreement with the simulation results [16]. It can be found that this curve has multiple sets of different defocus distances corresponding to the same distance *L*. It is contributed to geometric laser beam variations or fluctuations. Therefore, as stated in the following section, the effects of laser fluctuations could be suppressed by using the proposed data compensation algorithm.

### 3.2. Proposed Data Compensation Algorithm

Optics-based autofocusing microscopes are often used for the measurements at the micrometer level. Therefore, small noises may cause large errors in the measurement results, for example, just like the results of Figure 8. The causes of the errors are usually laser disturbance and environmental factors. The most direct way to obtain the interference of various errors on the laser spot image is to start with the laser spot image itself. Any external noise will cause the “shape” of the spot image to change. Therefore, possible relationships can be found from analyzing the geometry of the captured laser spot image and the geometric laser beam variations or fluctuations.

**Figure 9:**
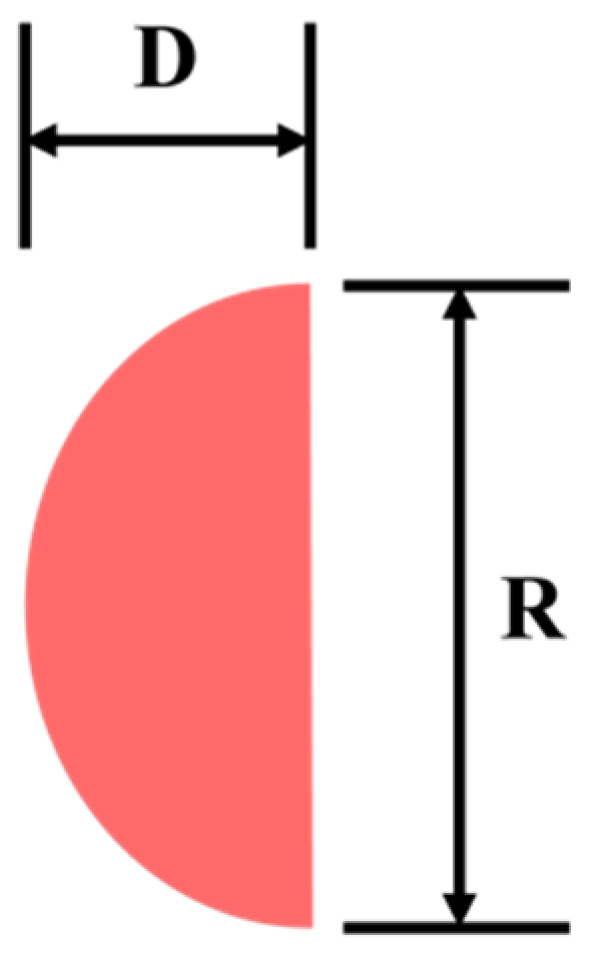
Definition of width *D* and height *R* for laser spot image.

First, we define the width and the height of the laser spot image as *D* and *R*, respectively, as shown in Figure 9.

**Figure 10:**
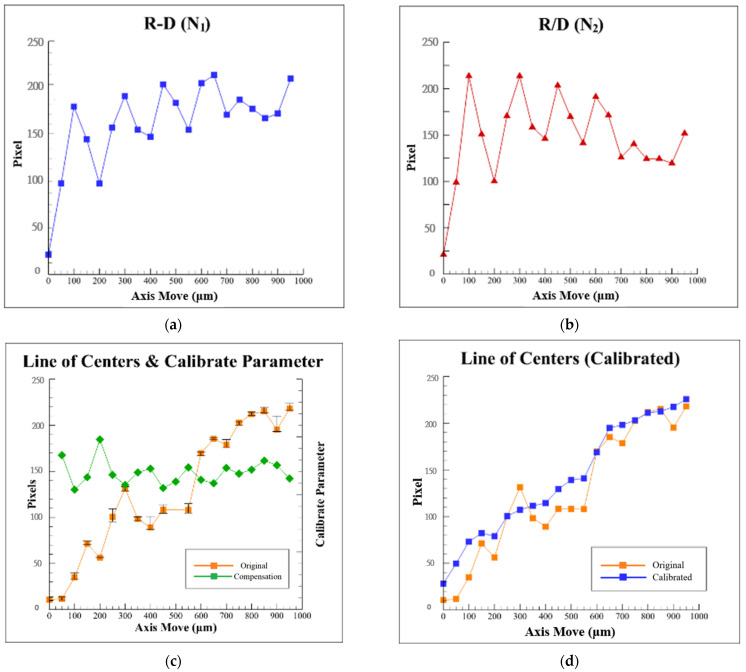
Measurement results of the proposed algorithm: variation of (**a**) value of *R*-*D*, (**b**) value of *R*/*D*, (**c**) compensation value, and (**d**) calibrated value with defocus distance, respectively.

Then we define $N_{1}=R-D,\;\;N_{2}=\frac{R}{D},$ divide *N*_2_ by *N*_1_, and multiply it by the corresponding value of the distance *L* to get the compensation value. These measurement results obtained for the variation of *N*_1_, *N*_2_, and *N*_2_*/N*_1_ with the defocus distance are illustrated in Figure 10a–c, respectively. Comparing these three curves in Figure 8 and Figure 10a,b, it can be found that the trends of these three curves are similar, which is no help for finding a pattern. From Figure 10c, it can be found that the trends of the two curves are opposite. The higher value in the original distance *L* is, the lower *N*_2_*/N*_1_ value will be, and vice versa. So, it is inferred that this value of *N*_2_*/N*_1_ can be used as a compensation value for the original distance *L* of the captured images to suppress the effects of geometric laser beam variations or fluctuations computationally.

This phenomenon is a significant finding in this study. Consequently, the following deduction can be obtained:

(5) $$\mathrm{Compensation}\;\mathrm{value}\;C=\mathrm{Original}\;\mathrm{distance}\;L\times \left[1+K\times \left(\frac{N_{2}}{N_{1}}\right)\right]$$

where *C* is the compensation number, *L* is the original distance, *K* ∈ ℚ, ℚ is the rational number, and $N_{1}=R-D,\;N_{2}=\frac{R}{D}$, and is mostly between 1 and 6. The worse the original accuracy of the system is, the higher the value of *K* will be.

Therefore, after multiplying the value of *N*_2_/*N*_1_ by six and adding to the original distance *L*, the calibrated distance *L* of the laser spot images in Figure 10d can be obtained by using the proposed data compensation algorithm. A total of six times results were used through the trial-and-error method for receiving the best value of *K*.

If we compare the two curves in Figure 10d, it can be found that the linearity of the calibrated curve is significantly improved to 97.81% from 91.77%, and it has demonstrated the feasibility of the proposed data compensation algorithm.

## 4. Experimental Results of Proposed Algorithm

**Figure 11:**
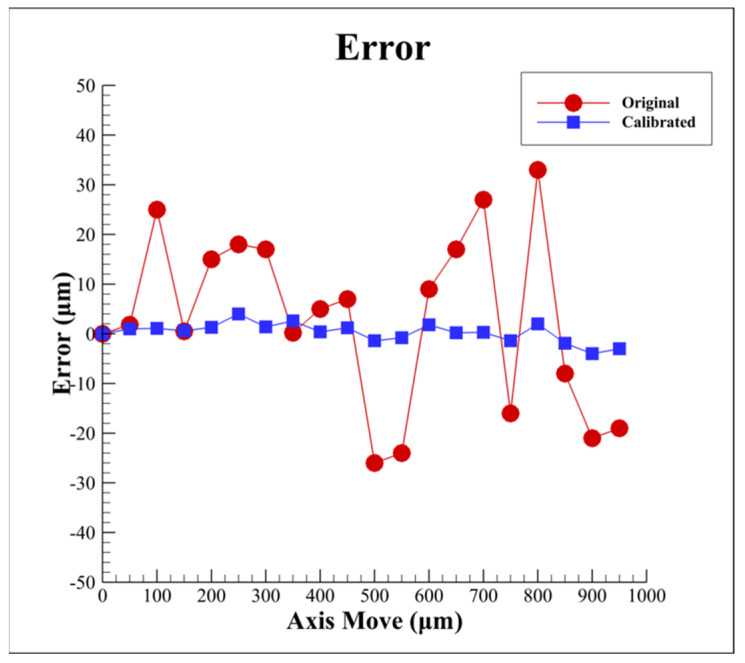
Experimental results for autofocusing accuracy with different defocus distances by using proposed algorithm and original distance *L* method, respectively.

As presented in Figure 4, to demonstrate the practical feasibility of the proposed algorithm, two sets of autofocusing experiments were carried out and compared using the proposed algorithm and the original distance *L* method [34], respectively, with different defocus distances *δ* (axis move). Figure 11 presents the experimental results for the autofocusing accuracy relative to the defocus distance *δ* (axis move), respectively. The original experimental results which are uncompensated have large errors and variations; however, the calibrated experimental results show the absolute value of the errors is about 1.5 to 2 μm. It is obvious that the autofocusing accuracy can be improved significantly by using the proposed algorithm.

**Figure 12:**
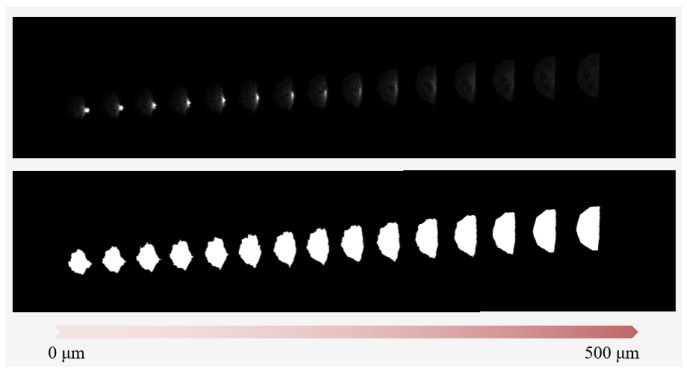
Experimental original and processed laser spot images on CCD_1_ with different defocus distances, respectively. The **top** one is original and the **bottom** one is processed.

As a means of comparing the conventional autofocusing microscope with the fair standards, the experiments in this study were conducted by using the same design parameters in [2]. Figure 12 shows experimental original and processed images of the laser spot on CCD_1_ for different defocus distances, respectively. The flowchart of the image processing in this study can be referred to our previous study [34]. It is noted that the real laser spot images are not a perfect semicircular, just like a semielliptical. Moreover, it can be found that the laser spot image has serious distortion near the in-focus position, so the original distance *L* method [34] cannot work well here.

**Figure 13:**
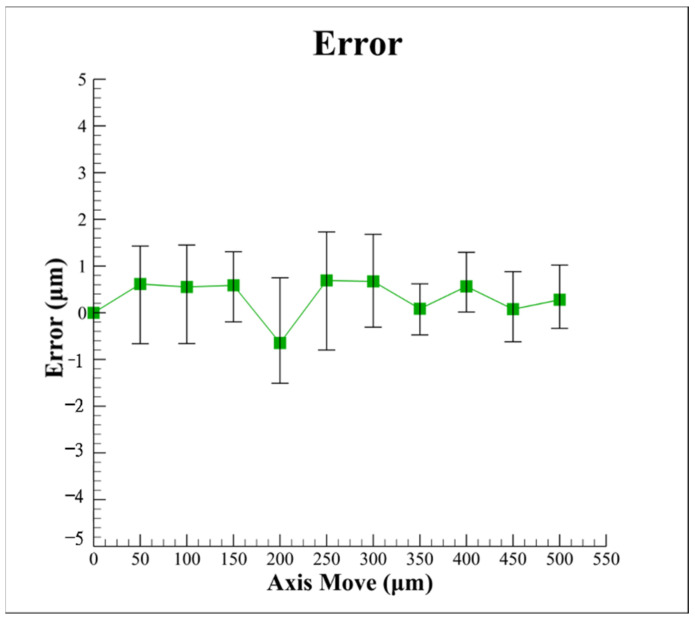
Experimental results for autofocusing accuracy with different defocus distances by using proposed algorithm.

Figure 13 illustrates the experimental autofocusing accuracy based on the proposed algorithm with different defocus distances *δ* (axis move). A total of five experiments were conducted for each defocus distance. As shown, the autofocusing accuracy by using the proposed algorithm can be improved to ≤1.5 μm, for which the range of average errors is between 0.5 μm and −1 μm and is better than that (≤2 μm) of the developed method by the rotating optical diffuser in [2]. In addition, the proposed algorithm does not add the hardware cost. In other words, the proposed algorithm for optics-based autofocusing microscopes can effectively improve the autofocusing accuracy and provides a promising method for suppressing geometric laser beam variations or fluctuations.

## 5. Conclusions

This present study has developed a set of algorithms that can quickly calculate the ideal ellipse center of the spot and effectively compensate the linearity of the focusing characteristic curve and has proposed a data compensation algorithm to improve the autofocusing accuracy and speed of the optics-based autofocusing microscope. By using a compensation value of the proposed algorithm, the variation of the image centroid position caused by geometric fluctuations of the laser beam is reduced. A prototype model has been constructed to assess the performance of the proposed algorithm.

According to the experimental results, the proposed algorithm has a higher accuracy of less than 1.5 μm when compared with the previous methods in [2] and [34]. Furthermore, the proposed method adds no other equipment to the system, which keep the system simple and saves the cost.

By reviewing the whole work presented in this present study, the contributions are concluded as follows:

- Calculating the center position of the ideal ellipse through the boundary of the light spot can improve the accuracy of the defocus distance calculation of the subsequent autofocus system.
- By using the proposed compensation algorithm, the linearity of the characteristic curve of the focusing system can be effectively improved, thereby achieving better accuracy of the optics-based autofocusing microscope.
- The proposed innovative algorithm can effectively remove the noise interference caused by environmental factors such as laser disturbance, instruments, and air temperature to compensate the measurement data based on the current spot shape, which not only reduces the equipment cost but also improves the system efficiency.

## Figures and Tables

**Table 1:** Design parameters of proposed autofocusing microscope.

Variable	Brand	Model
Laser	Thorlabs	HL6501MG
Beam Splitter	Thorlabs	BSW29 (50:50)
Biconvex Lens	Thorlabs	UB1945K
45° Red Dichroic Filter	Edmund	PBSW-633R
Sample	Thorlabs	Mirror
Objective Lens	Olympus	*f*_0_ = 18 mm
CCD_1_, CCD_2_	Basler	5472 px × 3648 px, 17 fps
Infinity-Corrected Optics System	Navita	1-60255
Motor	PI	UPL120 stroke of 13 mm

## Data Availability

Not applicable.
